# Hyaluronic Acid-Based Dynamic Hydrogels for Cartilage Repair and Regeneration

**DOI:** 10.3390/gels10110703

**Published:** 2024-10-30

**Authors:** Mingshuo Zhang, Qianwen Ye, Zebo Zhu, Shuanglian Shi, Chunming Xu, Renjian Xie, Yumei Li

**Affiliations:** 1 School of Medical Information Engineering, Gannan Medical University, Ganzhou 341000, China; zhangmingshuo@gmu.edu.cn (M.Z.); yeqianwen@gmu.edu.cn (Q.Y.); zhuzebo@gmu.edu.cn (Z.Z.); shishuanglian@gmu.edu.cn (S.S.); 2 Jiangxi Provincial Key Laboratory of Tissue Engineering (2024SSY06291), Gannan Medical University, Ganzhou 341000, China; xcm7604@gmu.edu.cn; 3 School of Basic Medicine, Gannan Medical University, Ganzhou 341000, China; 4 Key Laboratory of Prevention and Treatment of Cardiovascular and Cerebrovascular Diseases (Ministry of Education), Gannan Medical University, Ganzhou 341000, China

**Keywords:** hydrogels, hyaluronic acid, cartilage repair, cartilage regeneration

## Abstract

Hyaluronic acid (HA), an important natural polysaccharide and meanwhile, an essential component of extracellular matrix (ECM), has been widely used in tissue repair and regeneration due to its high biocompatibility, biodegradation, and bioactivity, and the versatile chemical groups for modification. Specially, HA-based dynamic hydrogels, compared with the conventional hydrogels, offer an adaptable network and biomimetic microenvironment to optimize tissue repair and the regeneration process with a striking resemblance to ECM. Herein, this review comprehensively summarizes the recent advances of HA-based dynamic hydrogels and focuses on their applications in articular cartilage repair. First, the fabrication methods and advantages of HA dynamic hydrogels are presented. Then, the applications of HA dynamic hydrogels in cartilage repair are illustrated from the perspective of cell-free and cell-encapsulated and/or bioactive molecules (drugs, factors, and ions). Finally, the current challenges and prospective directions are outlined.

## 1. Introduction

Hyaluronic acid (HA) is an important component of the extracellular matrix (ECM) of cartilage and is now a widely used biomaterial for tissue repair [1]. It was first isolated from the vitreous humor of the cow’s eye, and experimentally elucidated as an advanced polysaccharide composed of repeating D-glucuronic acid and N-acetylglucosamine [2]. Compared with the other natural polymers, such as gelatin, alginate, and chitosan, HA with its unique molecular structure and physicochemical properties in the body shows a variety of important physiological functions, such as anti-inflammation, lubrication of joints, promotion of wound healing, and also mediating its activity in cellular signaling [3,4,5,6]. Although HA alone is susceptible to degradation [7], the abundant modifiable groups such as carboxyl, hydroxyl, and N-acetylamino groups in HA allow us to introduce chemical or physical cross-links that can be targeted to improve the structure and properties of HA [8,9,10]. Specially, a dramatic improvement in the anti-degradation and/or mechanical properties of HA could be significantly improved when HA is designed to obtain hydrogels [11,12,13].

Hydrogels are highly hydrated polymer networks, which are often formed by chemical or physical cross-linking of polymer chains [14,15]. Benefiting from their high water content and flexibility similar to physiological conditions, hydrogels could mimic many aspects of natural cellular environment, and thus have been widely used for tissue repair and regeneration [16,17,18,19,20,21,22]. Conventionally, most hydrogels are prepared via stable, permanent, and covalent bonds [23], and therefore, the networks are static with limited dynamics. However, in living tissue, especially the ECM, the natural hydrogels not only provide the cells mechanical and structural support and serve as a reservoir for biochemical and biophysical cues, but also are able to remodel in response to cell activities or external stimuli so that the bioactive cues like growth factors or physical stimuli can be spatio–temporally presented to support cell survival, proliferation, and differentiation [24,25]. In this regard, the networks of ECM are dynamic, not static. Therefore, synthetic hydrogels, as artificial ECMs, would be able to mimic and recapitulate the dynamic characteristics of native ECMs when they are designed by reversible linkages, namely, dynamic hydrogels [26,27,28]. Unlike conventional static hydrogel, dynamic hydrogel is crosslinked by reversible covalent bonds (such as dynamic boronate ester bonds), physical or supramolecular interactions (such as hydrogen bonding and host-guest interactions), which can be de-crosslinking and subsequently re-crosslinking spontaneously by external stimuli, and therefore, dynamic hydrogel presents similar chemical and biophysical properties to living tissue, which is beneficial for cells and reshapes the surrounding microenvironment [29,30]. Recently, emerging work has demonstrated the unique advantages of dynamic hydrogels in tissue repair and regeneration [31,32,33,34,35], especially the HA-based dynamic hydrogels with good degradability without toxic degradation products, excellent biocompatibility, and good flexibility similar to the natural tissue [36].

Articular cartilage is a smooth and elastic tissue that reduces friction cushions stresses between bones to maintain daily frictionless and normal movement; however, articular cartilage damage due to trauma and aging has become a common health issue and remains a big challenge in clinical settings due to the avascular, unnerved, no lymphatic nature of articular cartilage [37]. Without timely intervention, the articular cartilage damage area gradually expands and develops to the most common joint disease, osteoarthritis (OA), which affects millions of people worldwide [38]. Currently, the commonly used methods to repair articular cartilage include microfracture surgery, osteochondral transplantation, autologous chondrocyte transplantation technology, and matrix-induced chondrogenesis technology [39,40,41]. However, significant limitations, such as scarcity of chondrocyte source, immune response or rejection, the challenge of large areas of cartilage damage, and the regenerated fibrocartilage, not articular cartilage, imperatively trigger to develop methods to evade these limitations to effectively promote physiological regeneration [42]. As hydrogels are able to mimic the structural properties of natural ECM, they can effectively support the movement of loaded cells and drugs in three-dimensional spaces and maintain their activity. In addition, hydrogels contain a large amount of water, which can increase the load-bearing capacity during movement [43] while reducing the friction coefficient of articular cartilage [44,45]. In addition, the structural composition of hydrogels is sufficient to form a scaffold to provide appropriate mechanical properties. The current structural design of hydrogels allows hydrogels to possess properties such as injectability and adhesion [46], which provides additional advantages when used for cartilage repair and regeneration. HA-based hydrogels were chosen because HA can improve cartilage ECM deposition, promote cell adhesion and proliferation, and improve the lubricity of cartilage borders compared to other polymer hydrogels [47,48,49]. Moreover, it has been shown that HA can recognize clusters of differentiation 44 (CD44) to participate in cartilage regeneration during cartilage repair [50]. Given articular cartilage itself is a dynamic hydrogel (with water content up to 80%) and HA as one of the main components, the emergence of HA-based dynamic hydrogels has brought about more opportunities for cartilage repair and regeneration [17,51,52].

**Scheme 1:**
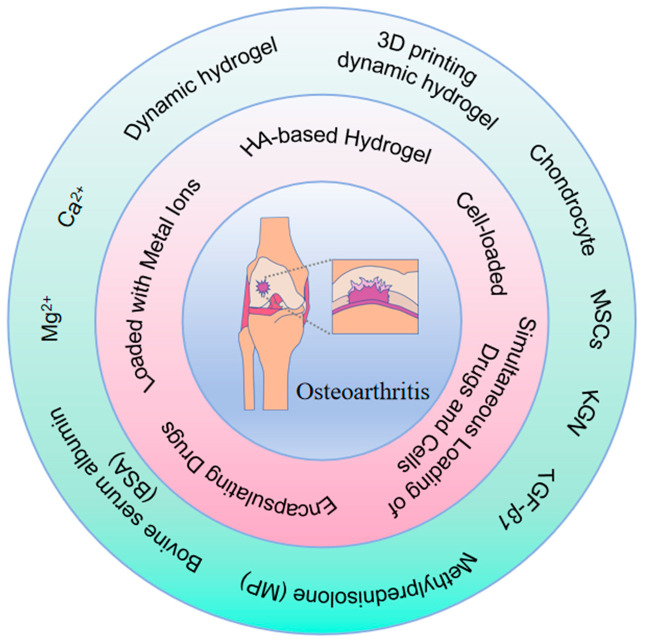
Research methods for the treatment of articular cartilage and regeneration.

In this review, we would like to summarize the recent advances of HA-based dynamic hydrogels and their application in articular cartilage repair and regeneration (Scheme 1). The various cross-linking methods to prepare HA-based dynamic hydrogels are firstly overviewed, and subsequently, the applications in cartilage repair and regeneration are summarized from the perspective of cell-free and cell-encapsulated and simultaneously, with or without bioactive cues, such as growth factors and metal ions. This focuses on the repair of articular cartilage by hydrogels containing different loadings (Table 1). Finally, the current challenges and future prospects are presented.

## 2. Cross-Linking of Dynamic HA-Based Hydrogels

As HA is a component of the human body and has a large number of groups that can be modified, such as carboxyl groups, aldehyde groups, etc., the modification of HA using the appropriate techniques can result in hydrogels with different structures, activities and functions [46,59]. Since hydrogels synthesized from a single HA are weak in all aspects, in order to better act on the lesion, different modifications are needed to make the HA-based hydrogels emphasize different effects. This section then summarizes the cross-linking of dynamic HA derivative hydrogels and the intended properties of this hydrogel.

### 2.1. Chemical Cross-Linking

#### 2.1.1. Schiff Base Bonds

**Figure 1:**
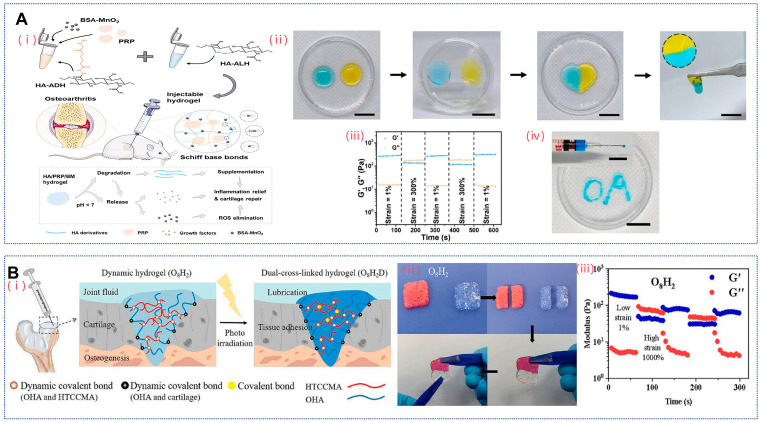
HA hydrogels based on Schiff base bonding. (**A**). (**i**) HA/PRP/BM injectable hydrogel prepared by Schiff base reaction synergistically treats OA by viscosity enhancement, eliminating ROS, relieving inflammation, and promoting cartilage repair. (**ii**) Images of hydrogel gelation and self-healing process. (**iii**) Step strain test of hydrogel with fixed shear frequency of 1 Hz at 37 °C. (**iv**) Hydrogel can be injected through a 29-gauge needle without clogging. Reproduced with permission from ref. [56], copyright, Elsevier. (**B**). (**i**) Preparation of double cross-linked OHA/HTCCMA hydrogel for articular cartilage repair. (**ii**) The O_8_H_2_ hydrogel was cut in half and re-attached. (**iii**) Alternate step strain test of O_8_H_2_ hydrogel. Reproduced with permission from ref. [53], copyright, Elsevier.

Schiff base bonds, also known as imine bonds, are produced by reversible condensation reactions between primary amines and active carbonyl groups, such as aldehydes and ketones. The dynamic equilibrium of the Schiff base bond is therefore utilized to enable the preparation of self-healing hydrogels. In view of this, Zhou et al. applied the Schiff base bond between OHA and adipic dihydrazide grafted hyaluronic acid (HA-ADH) to construct dynamic hydrogels (Figure 1A) [56]. Due to the nature of Schiff bases, the hydrogels were self-healing, and their hydrogels that changed to the sol–gel state at high strains (300%) could return to the gel state at low strains (1%). In addition, both polymers readily formed hydrogels in PBS at pH 7.4 with a short gel time of 20 s and a storage modulus of about 600 Pa. In this experiment, the hydrogels also contained platelet plasma (PRP), whose incorporation led to an increase in the gel time and a decrease in the storage modulus of the hydrogels. This is because when PRP particles are mixed with HA-ALH molecules, the amino group in the protein immediately reacts with the aldehyde group on HA-ALH to form an imine bond, consuming some of the aldehyde group that reacts with HA-ADH. As a result, the reaction rate slows down, the crosslinked network becomes smaller, and the mechanical strength of the hydrogel decreases, leading to faster degradation as well. However, this structure facilitates its injectability and releasability. This self-healing and injectability gives hydrogels the potential to be injected within weight-bearing joints to improve lubrication and protect cartilage.

Although the aforementioned hydrogels provide good lubrication, cartilage injuries are often less regular in shape and therefore also require hydrogels that can be anchored to the wound to maintain a stable cure. To address this issue, Qiu et al. prepared a double crosslinked hydrogel using OHA and N-(2-hydroxypropyl)-3-trimethylamine chitosan chloride (HTCC) methacrylate (HTCCMA) as raw materials (Figure 1B) [53]. This hydrogel formed a dynamic hydrogel by the Schiff base reaction of the amino group in HTCCMA with the aldehyde group in OHA. The hydrogel affixed within 1 min, and the hydrogel could not be easily separated by pulling with tweezers. In addition, the hydrogel was also able to completely transform between the sol and gel states under repeated small (1%) and large (10,000%) strains, showing its excellent self-healing properties, which is inseparable from the contribution of the dynamic crosslinking structure in the hydrogel. The methacrylate groups in the HTCCMA were then crosslinked by light irradiation, and the storage modulus of the doubly crosslinked hydrogel was significantly increased to about 1750 Pa compared with that of the dynamic hydrogel alone, resulting in a significant enhancement of its mechanical properties. Additionally, the Schiff base reaction between the aldehyde group in OHA and the amino group of glycosaminoglycan on the cartilage surface, along with electrostatic interactions and hydrogen bonding between the hydrogel and cartilage surface, can effectively immobilize OHA, thereby conferring moderate stability to tissue adhesion properties of the hydrogel [53]. It is worth noting that a mild adhesion of the hydrogel to cartilage may promote joint lubrication; however, excessive adhesive strength could hinder diffusion and distribution of the hydrogel within the joint cavity post-injection, potentially impacting normal patient ambulation.

Schiff base-bonded cross-linked HA-based dynamic hydrogels have been widely used in the biological field due to their ease of use, mild reaction conditions, and relatively easy access to gel-forming materials [60,61,62]. In this aspect of cartilage repair, this type of hydrogel has good self-healing, injectability, and adhesion properties, thus providing a promising solution for degenerative cartilage diseases such as OA.

#### 2.1.2. Acylhydrazone Bond

**Figure 2:**
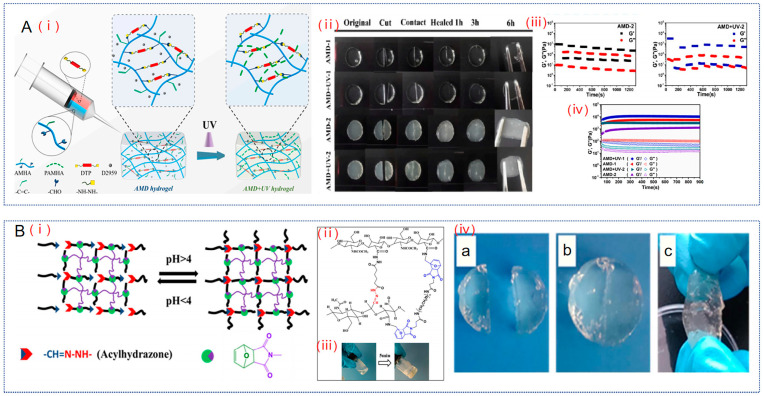
HA hydrogels based on acylhydrazone bonds. (**A**). (**i**) Schematic diagram of the hydrogel. (**ii**) Photograph of the healing process after cut disruption. (**iii**) Alternating step strain scanning curves of AMD-2 hydrogel and AMD + UV-2 hydrogel. (**iv**) Gel formation curves. Reproduced with permission from ref. [63], copyright, Elsevier. (**B**). (**i**) Diagram of cross-linking mechanism of DN network. (**ii**) Scheme of the double cross-linking process (dynamic covalent acylhydrazone bonds labeled in red and Diels–Alder bonds chemically labeled in blue). (**iii**) Gel behavior of double crosslinked hydrogels. (**iv**) Self-healing properties of DN hydrogels (**a**–**c**). Reproduced with permission from ref. [65], copyright, ACS Publications.

The acylhydrazone bond is an imine bond generated by the reaction of a hydrazide and an aldehyde group, and its stability is superior to that of normal imine bonds. Although the self-healing efficiency of the Schiff base-based HA hydrogel was 100% after 10 min of healing, its tensile stress was only 1 kPa [16]. In contrast, the acylhydrazone bond-based HA hydrogel not only had highly efficient self-healing properties but also an increased tensile stress. For example, Zhang et al. prepared a self-healing HA-based hydrogel with enhanced strength by dynamic acylhydrazone bonding between aldehyde-modified sodium maleate hyaluronate and 3,3′-dithiopropionylhydrazine and photopolymerization of the maleic acid moieties in a hydrogel network (Figure 2A) [63]. The storage modulus (G′) of this hydrogel could exceed the loss modulus (G″) within 50 s, indicating its rapid gelation ability. Moreover, the hydrogel can be cut in half and fully fused into a complete hydrogel after 6 h at room temperature, and this self-healing hydrogel is strong enough to withstand tensile forces in the direction perpendicular to the contact surface. In addition, the original compressive strength of both the simple dynamic hydrogel and the double crosslinked hydrogel after light irradiation exceeded 100 kPa, which was significantly higher than that of the Schiff base hydrogel, indicating that its mechanical properties were enhanced. Based on these results, it can be found that acylhydrazone-bonded HA hydrogels possessed higher mechanical strength compared to Schiff base-based hydrogels, and their self-healing properties were not affected.

In addition, another characteristic of acylhydrazone bonds is pH responsiveness; when the pH falls below 4, the acylhydrazone bond breaks, resulting in the transition of the hydrogel from a gel to a sol [64]. In contrast, when OA occurs, the environment in the joint cavity is acidic, and consequently, the simple acylhydrazone bond hydrogel is likely to fracture. Therefore, to solve this problem, Yu et al. designed and prepared a double cross-linked network hydrogel by introducing the dynamically reversible acylhydrazone bond formed by acylhydrazones and aldehydes into the cross-linked network of the Diels–Alder bonding reaction (Figure 2B) [65]. Because the DA-crosslinked hydrogel had good cell encapsulation, it maintained the structural integrity and mechanical strength of the hydrogel under a physiological environment. The self-repair process of acylhydrazone bonds has been reported to occur without any external intervention at ambient temperatures [66]. So, the acylhydrazone bond becomes a switch to control the cross-linking density of the network. In addition, it is exciting that the hydrogel contains aldehyde groups that can further covalently bind Schiff base with local cartilage histamine and in this way can act as an adhesive to cartilage tissue. All in all, the hydrogel has a more stable structure and higher compression modulus both in acidic solution and in a physiological environment.

Acylhydrazone bonds possess higher mechanical strength compared to imine bonds, and therefore, better results can be obtained when applied at the joints. However, the pH responsiveness of acylhydrazone bonded HA-based hydrogels also needs to be considered when applied to OA. Moreover, acylhydrazone bonds are formed through strong redox reactions and free radical generation, which are prone to cytotoxicity [64]. Therefore, the cytotoxicity of acylhydrazone HA-based hydrogels also needs to be addressed if they are to be useful in the clinic.

#### 2.1.3. Borate Bond

**Figure 3:**
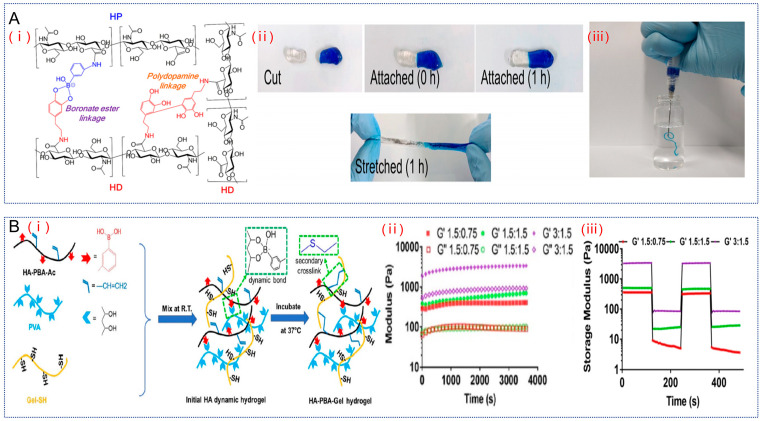
HA hydrogel based on borate bonding. (**A**). (**i**) A scheme of a pH-controlled cross-linked hydrogel system. (**ii**) Self-healing properties of the hydrogel verified by tensile tests. (**iii**) Injectability test of hydrogel (with methylene blue) in a single syringe system. Reproduced with permission from ref. [67], copyright, ACS Publications. (**B**). (**i**) Schematic diagram of dynamic HA hydrogel crosslinked with gelatin formation. (**ii**) Variation in storage modulus (G′) and loss modulus (G″) with time for different hydrogels. (**iii**) Shear recovery properties of gelatin cross-linked dynamic HA hydrogels. Reproduced with permission from ref. [71], copyright, IOP Publishing.

The borate bond is formed by the interaction of the three oxygen atoms of boron in the borate and the hydrogen atom of the alcohol group. It can dissociate at acidic pH and is reversible. Thus, the dynamic equilibrium of the borate bond makes it more useful in drug delivery. For example, Lee et al. used phenylboronic acid grafted on hyaluronic acid (HA-PBA) crosslinked with dopamine grafted on Dop-HA to obtain a dynamic boronated ester-based hydrogel (Figure 3A) [67]. The network structure of this hydrogel was composed of borate and polydopamine bonds and the pH of HA-PBA and Dop-HA was carefully and continuously controlled, resulting in the preparation of an injectable gel optimized for a single syringe system that works around physiological pH and reduces post-injection pain due to pH differences. The hydrogel healed in about 1 h after being cut, and did not break after a single pull was applied to it. Moreover, the hydrogel is easy to inject through a syringe needle and is in the form of a gel when injected into water. Therefore, the hydrogel has good self-healing properties and is injectable with a single needle. As a result, it can be injected into the affected area through the gradual degradation of the hydrogel to achieve the gradual release of the drug, it can offer a continuous treatment of the affected area.

Borate bonding has the additional feature that it can effectively scavenge H_2_O_2_ molecules without a catalyst and without the formation of cytotoxic by-products [68]. Therefore, a series of novel hydrogels with antioxidant properties has been developed on the basis of borate bond-based dynamic hydrogels [69,70]. For example, Shi et al. crosslinked HA-PBA with thioglycolic-containing polyvinyl alcohol (PVA) to obtain a borate ester-based hydrogel (Figure 3B) [71]. This hydrogel was formed by the formation of borate ester dynamic bonds between the PBA groups of the modified HA and the diol groups in PVA, and it could undergo rapid gelation in less than 15 s at room temperature. It was found through recovery tests that when the hydrogel was subjected to a strain of 500%, all storage modulus G′ values immediately decreased significantly. Once the strain was restored to the initial low strain, the G′ values of this hydrogel rapidly recovered back to almost the initial G′ values. This proves that it has good self-healing property. Moreover, secondary cross-links can be formed between the modified HA-grafted acrylate and the free sulfhydryl groups of the vulcanized gelatin to enhance the stability. In the secondary cross-linking between HA and gelatin, the sulfhydryl ratio plays an important role in regulating the modulus of the hydrogel. The more sulfhydryl groups, the larger the G′ of the hydrogel will be. In the hydrogel with 3:1.5 gelatin compared to HA, its G′ increased over time from an initial 1830 Pa to 3370 Pa within 1 h. Its antioxidant property was observed by staining the cells with an H_2_O_2_ responsive probe, and it was seen that there was no statistically significant difference in the average fluorescence intensity of the control group and the hydrogel group, which suggests that the hydrogel can effectively remove H_2_O_2_ from the culture medium and reduce the oxidative stress in cultured cells in vitro.

In summary, borate bonds have many advantages for the construction of dynamic hydrogels. For example, the diol moiety is present in most natural polysaccharides and is readily available without the need for complex synthetic processes. Furthermore, borate bonds have excellent self-healing properties and are antioxidant, properties that allow for sustained action and the elimination of inflammation when applied to OA. However, despite the many advances in these hydrogels, the reaction conditions between the boric acid and the diol groups are harsh and far from clinical application.

#### 2.1.4. Disulfide Bonds

In recent years, the disulfide bond of the bionic protein structure has become a hot research topic, which usually consists of two thiol groups coupled together and is a relatively stable covalent bond [72,73]. In HA-based hydrogels, it is generally necessary to sulfurize HA to obtain sulfide hyaluronic acid (HA-SH) before cross-linking it with other substances containing thiol groups, in addition to the fact that the sulfur groups of HA-SH can cross-link on their own to form in situ gels.

**Figure 4:**
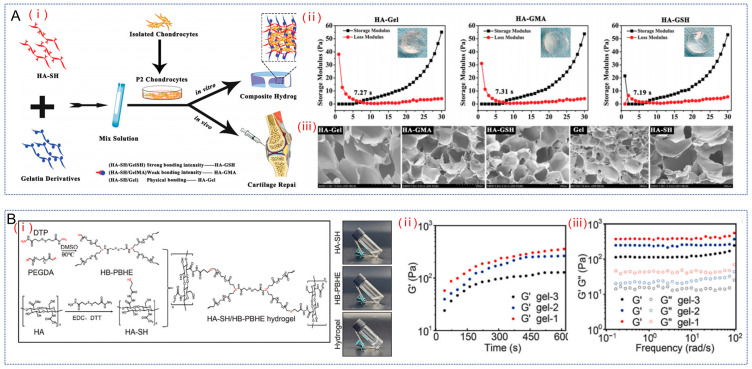
HA hydrogels based on disulfide bonds. (**A**). (**i**) Preparation and application process of composite hydrogels. (**ii**) Macroscopic morphology and gelling time measured by rheological test. (**iii**) Internal microstructure of the hydrogel measured by scanning electron microscopy. Reproduced with permission from ref. [74], copyright, Royal Society of Chemistry. (**B**). (**i**) Schematic diagram of the synthesis process of HB-PBHE, HA-SH, and the generated HA-SH/HB-PBHE hydrogel. (**ii**) Dynamic time-scanning measurements. (**iii**) Variation in G′ and G″ with angular frequency for HA-SH/HB-PBHE hydrogel. Reproduced with permission from ref. [75], copyright, Elsevier.

For example, Wang et al. prepared in situ gels of HA-SH (HA-Gel) as well as biomimetic composite hydrogels based on weak (HA-GMA) and strong (HA-GSH) disulfide bond strengths of HA-SH and modified gelatin derivatives (Gel-SH) (Figure 4A) [74]. It can be found that the strong disulfide bonding helps to maintain a more homogeneous pore structure with improved water retention and resistance to degradation and is more favorable for chondrocyte proliferation and maintenance of hyaline cartilage phenotype. In addition, a hydrogel crosslinked with HA-SH and hyperbranched poly (β-hydrazide ester) (HB-PBHE) was prepared by Tan et al. in order to reduce the loss when delivering exosomes into the pericardium (Figure 4B) [75]. The HA-SH component effectively replicates the composition and hardness of the pericardial fluid, thereby preserving the structural integrity of the pericardium. Additionally, the disulfide bonds present in its molecular structure not only enhanced biodegradability, but also confer unique capabilities for scavenging free radicals.

Overall, HA-SH can be used as a stand-alone material to form in situ hydrogels via disulfide bonds, and the strength of the disulfide bonds can also affect the mechanical strength of the hydrogel. Although disulfide bonds are similar in structure to human proteins and have good biocompatibility, HA-based hydrogels are less commonly used, perhaps because the disulfide bonds are ‘weak’ compared to other cross-linking disulfide bonds, which are easier to break and less controllable.

### 2.2. Physical Crosslinking

Physically crosslinked hydrogels have a wide range of biomedical applications because their assembly dynamics are more similar to biological systems. Physically crosslinked dynamic hydrogels are produced by non-covalent interactions, which include hydrogen bonding, metal ligand coordination, host-guest interactions, and hydrophobic interactions. Here, we focus on the first two.

**Figure 5:**
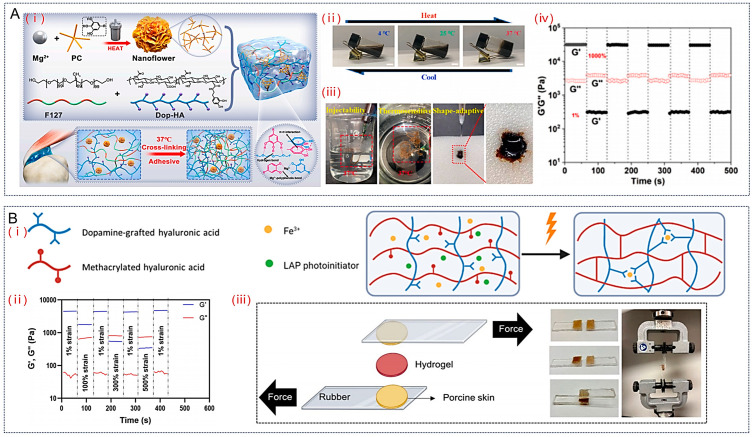
Physically crosslinked dynamic HA hydrogel. (**A**). (**i**) Schematic design of an injectable composite hydrogel. (**ii**) Macroscopic photographs of the hydrogel at different temperatures. (**iii**) Injectability, thermal sensitivity, and shape adaptation. (**iv**) Rheological recovery behavior of composite hydrogels. Reproduced with permission from ref. [58], copyright, KeAi. (**B**). (**i**) Schematic diagram of dual network hydrogel. (**ii**) Self-healing behavior of the samples when low (1%) and high (100%, 300%, and 500%) strain cycles are applied alternately. (**iii**) Schematic of adhesion measurement. Reproduced with permission from ref. [76], copyright, Nature Portfolio.

Compared to chemical cross-linking, physically cross-linked dynamic hydrogels possess superior shear-thinning properties and therefore good injectability, allowing for a more efficient delivery of cells or bioactive molecules to the target site via minimally invasive procedures, as well as easy filling of large and irregular complex defects. Li et al. used hydrogen bonding to cross-link Dop-HA and F127 to obtain a hydrogel with good injectability, thermal sensitivity, and adhesion (Figure 5A) [58]. The storage modulus of this hydrogel was lower than the depletion modulus when it received a strain of 1000%. When the strain is recovered to 1%, the G′ and G″ values can again both recover or exceed their initial values. This indicates that the hydrogel has a fast and efficient self-healing ability. In addition, the hydrogel remained in a liquid state at 4 °C or 25 °C, while at 37 °C, the hydrogel cured. The hydrogel also possesses good adhesion properties due to the presence of catechol groups on the dopamine moiety. All in all, this hydrogel can be injected into the treatment site from outside the body with a syringe, it solidifies with increasing temperature upon entry into the body, and it does not flow to other tissues due to the presence of dopamine. These features facilitate the injection process and allow the hydrogel to fit seamlessly into irregularly shaped injury sites.

Due to the good adhesion properties of the catechol group of dopamine, Nejati et al. used dopamine grafted oxidized hyaluronic acid (DAHA) crosslinked with hyaluronic acid methacrylate (HAMA) via metal ligand coordination to form hydrogels (Figure 5B) [76]. This is due to the fact that dopamine contains catechol, which is a functional group with chelating properties toward Fe^3+^, thus rapidly forming a physical cross-linking network through the coordination of Fe^3+^ and dopamine and endowing the hydrogel with rapid gelation and self-healing properties [77]. In addition, in order to improve the mechanical properties, the HA chains in the hydrogel were modified with methacrylate groups, which further strengthened the cross-linking of the physical network formed spontaneously under UV light irradiation [76]. The resulting hydrogel could rapidly form a self-healing microporous adhesive scaffold with a compression modulus of 29.4 kPa and an adhesion strength of 12.8 kPa within 6 s. The resulting hydrogel could be used as a self-healing microporous adhesive scaffold with an adhesion strength of 12.8 kPa.

Injectable hydrogels are biomaterials that possess minimal invasiveness and the capability to fill irregularly shaped defects, thus rendering them suitable for application in regenerative medicine. [78,79,80]. In order to be considered suitable for in situ injection, hydrogels must meet a number of material design requirements. Thus, rapid gelation is required to prevent leakage of the pre-gel into the surrounding area, and it also needs to be self-healing to ensure that it can return to its original properties after being subjected to high-intensity damage [81]. But translating this hydrogel into clinical applications requires further research. These additional studies will provide valuable insights into its safety, efficacy, and long-term performance, thus advancing its clinical application.

### 2.3. Multiple Crosslinking

**Figure 6:**
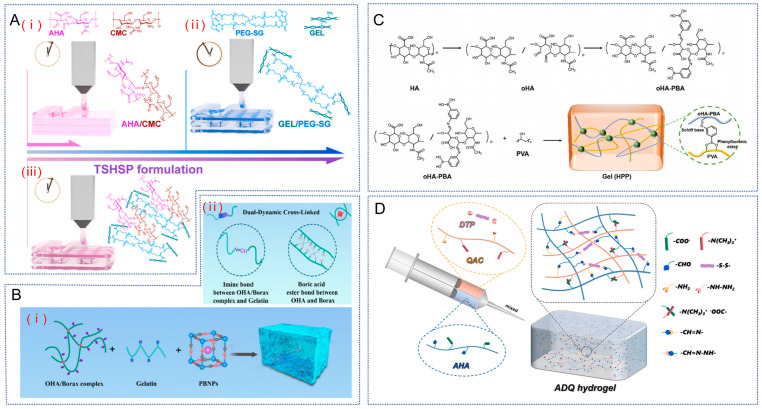
HA hydrogels based on multiple dynamic cross-links. (**A**). (**i**) Microstructural fusion of fast gel AHA/CMC hydrogel print constructs based on dynamic imine bonds. (**ii**) Preparation of AHA/PEG-SG slow gels through the formation of stable amide bonds. (**iii**) TSHSP hydrogels prepared by complementary AHA/ CMC and GEL/PEG-SG. Reproduced with permission from ref. [82], copyright, Elsevier. (**B**). (**i**) Preparation scheme of PBNPs@OBG hydrogels. (**ii**) Schematic structure of a hydrogel consisting mainly of imine and borate bonds. Reproduced with permission from ref. [19], copyright, BMC. (**C**). Scheme for the formation of double dynamic bond crosslinked hydrogels (HPP). Reproduced with permission from ref. [54], copyright, Wiley. (**D**). Schematic diagram of ADQ hydrogel preparation. Reproduced with permission from ref. [85], copyright, Elsevier.

Currently most hydrogels are formed by one dynamic cross-linking, while some hydrogels are obtained as double cross-linked hydrogels with one dynamic and one static in order to improve the mechanical strength. Therefore, in order to adapt to different roles, hydrogels can also have two dynamic crosslinks. For example, Chen et al. wanted to obtain a low stiffness, high water retention, and high permeability hydrogel for use as a bio-ink, and therefore used the Schiff base reaction between aldehyde hyaluronic acid (AHA) and n-carboxymethyl chitosan (CMC) to form a fast dynamic cross-linking, while the formation of an acylhydrazone bond between gelatin (GEL) and tetra-armed poly (ethylene glycol) succinimidyl glutarate (PEG-SG) was used to obtain a slow stabilizing crosslink, resulting in a dynamic double crosslinked hydrogel (Figure 6A) [82]. This hydrogel has good viscoelastic and self-healing properties and can be printed within a short period of time after preparation and can maintain structural stability during the printing process.

The Schiff base reaction, as one of the simplest and most frequently used modes of dynamic hydrogel synthesis, has a wide range of applications in double cross-linking. For example, Yang et al. designed an injectable hydrogel with rapid gelation and self-healing properties through double dynamic bond crosslinking between OHA, borax, and gelatin (Figure 6B) [19]. The hydrogel was formed by first forming a borate ester bond from borax and OHA, and the obtained product was then combined with gelatin to obtain a Schiff base bond. Many studies have demonstrated that both borate and Schiff base bonds are acid-sensitive bonds [83,84]. Therefore, the hydrogel presented in Figure 6B lasts for about 30 days in PBS at pH 7.4, whereas in PBS at pH 6.5 the hydrogel degrades completely within 24 days, suggesting that it has pH-responsive degradation properties in vitro.

Lei et al. also synthesized a hydrogel that is injectable, self-healing and provides lubrication via Schiff base bonds and borate ester bonds [54]. In this hydrogel, the amino group in 3-aminophenylboronic acid and the residual aldehyde in OHA synthesized the Schiff base bond, and then the phenylboronic acid in 3-aminophenylboronic acid modified OHA and hydroxyl group in hydroxyl-containing poly (vinyl alcohol) synthesized the phenyl borate bond (Figure 6C). The phenyl borate bond has ROS responsiveness and excellent ROS scavenging ability, so the hydrogel helps to reduce inflammation and relieve joint pain and swelling. And the hydrogel can be dispensed with a syringe, and it also quickly returns to the gel state when the hydrogel is dispensed from the needle, indicating that it has good injectability.

In addition to this, there are hydrogels formed using four types of dynamic cross-linking. Yang et al. [85] started with dynamic Schiff base bonding between -NH_2_ of quaternary ammonium chitosan (QAC) and –CHO of AHA, while electrostatic interactions are also generated between –N (CH_3_)^3+^ of QAC and –COO^-^ of AHA. In addition, reversible acylhydrazone bonds can be formed using the disulfide-containing 3, 3′- dithiobis (propionyl hydrazide) (DTP) of –NH_2_ with the –CHO of AHA (Figure 6D). The resulting hydrogel then includes four dynamic covalent bonds and thus has rapid gelation and an excellent self-healing ability: the hydrogel can undergo a rapid sol–gel transition within 10 s and can heal completely within 1 h after being disrupted.

Overall, whether the hydrogel contains several dynamic cross-links, the ultimate goal is that this hydrogel can have self-healing ability. In biomedical engineering, HA hydrogels with self-healing abilities have attracted much attention because of their inherent biocompatibility, biodegradability, viscoelasticity, and good ability to regulate cellular behavior. Therefore, it is also hoped that hydrogels with better properties can be developed and widely used in biomedical fields.

## 3. Cartilage Repair and Regeneration

**Figure 7:**
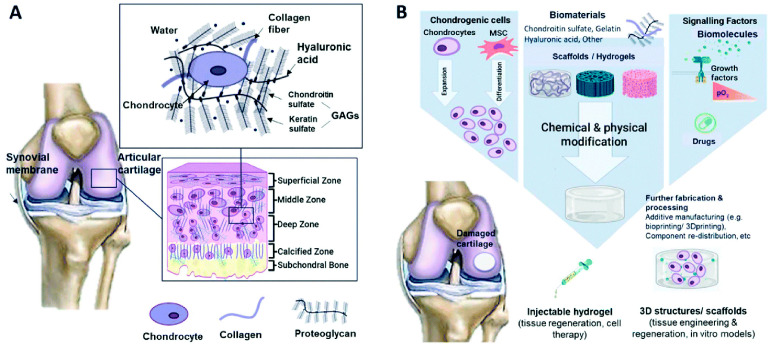
Illustration articular cartilage structure and composition (**A**) and the tissue engineering method for cartilage repair and regeneration (**B**). Reproduced with permission from reference [86], copyright, RSC Publishing.

Articular cartilage, as an important component of the human body, plays a crucial role in providing mechanical support, cushioning impact, and reducing friction. Articular cartilage is a highly specialized connective tissue composed of ECM and chondrocytes, as shown in Figure 7 [86]. Whereas in natural cartilage tissue, chondrocytes are in a dynamic and hydrated three-dimensional ECM microenvironment [87], the limitations of cartilage self-repair can be overcome by developing cellular scaffolds that mimic the complex structure of cartilage tissue. Hydrogels are a suitable choice, especially those based on natural polysaccharides such as HA, chitosan, and chondroitin sulfate, a macromolecular structure similar to the tissue ECM [88], which mimic many of the characteristics of the tissue ECM and have the potential to guide cellular behaviors during tissue regeneration. We therefore hope that we can provide relief and treatment through hydrogels applied to the injured area, and this section summarizes the more effective HA-based dynamic hydrogels that are currently available for treatment.

### 3.1. HA-Based Hydrogel

Clinical research has demonstrated that HA can improve joint lubrication and improve the proliferation of chondrocyte and matrix deposition in a dose-dependent manner [89]. Zhu et al. prepared hydrogels of hydrazine-modified elastin and aldehyde-modified HA with variable HA concentration by dynamic hydrazone bonding reaction [90]. It can be found that with the increase in HA concentration, the gene expression of cartilage-specific markers will be elevated and the deposition of glycosaminoglycans will be increased, while controlling the hydrogel stiffness constant. The hydrogel system formed by oxidized pectin grafted with HA-hexanediol dihydrazide and oligopeptide G4RGDS by Chen et al. also demonstrated that HA hydrogels are suitable for chondrocyte proliferation and growth [91].

**Figure 8:**
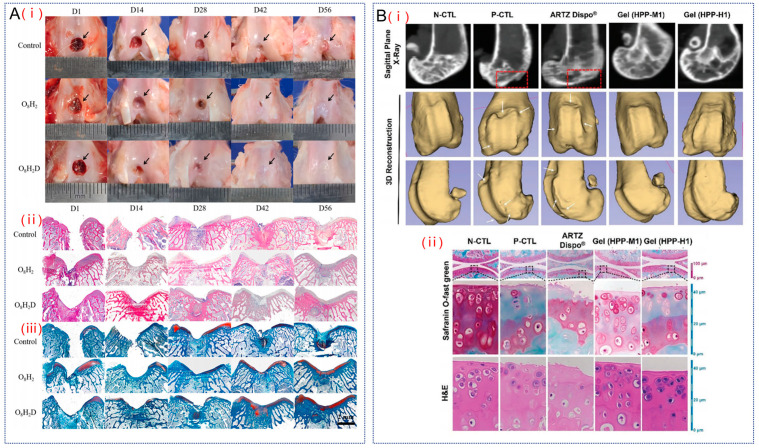
Dynamic HA hydrogel for the treatment of OA. (**A**). Cartilage repairing effect of double crosslinked OHA/HTCCMA hydrogel. (**i**) Photographs of the gross appearance of healed rabbit articular cartilage within 56 days. (**ii**) H and E staining, (**iii**) SO/FG staining images of cartilage tissue. Reproduced with permission from ref. [53], copyright, Elsevier. (**B**). In vivo therapeutic effect of intra-articular injection of double dynamic bond cross-linked hydrogel (HPP) on OA in mice. (**i**) CT reconstruction model of femoral condyle. (**ii**) Representative images of SF and H and E stained mouse joints. Reproduced with permission from ref. [54], copyright, Wiley.

However, conventional hydrogels cannot be anchored to the wound to maintain a stable cure, so Qiu et al. prepared a double cross-linked hydrogel with OHA and N-(2-hydroxypropyl)-3-trimethylammonium chitosan chloride methacrylate (HTCCMA) [53]. Due to the presence of a large amount of glycosaminoglycans in cartilage [92], it can react with the aldehyde group in the hydrogel OHA, and together with the electrostatic interactions and hydrogen bonding between the interfaces, it is possible to fix the hydrogel on the cartilage. Moreover, HA itself is one of the main components of synovial fluid in the joint cavity, so the HA in the hydrogel serves as a lubricant and can reduce the friction between tissues. In addition, quaternized chitosan has good antimicrobial properties and can inhibit trauma-induced wound infections [93], so the hydrogel also exhibits good antimicrobial properties, all of which contribute to attenuating the symptoms of OA. The hydrogel was also seen to promote cartilage regeneration and prevent cartilage damage compared to the control group in the animal femoral defect model experiment (Figure 8A). Overall, the hydrogel provides a promising solution for degenerative cartilage diseases such as OA

In addition to lubrication and antimicrobials, one of the etiological factors to be concerned with in the treatment of OA is high levels of reactive oxygen species (ROS). High levels of ROS cause oxidative stress, inducing cellular damage and death, and also induce the proliferation of inflammatory cytokines and cellular chemotaxis, further exacerbating the inflammatory reaction. In fact, under normal physiological conditions, it is essential to maintain normal cell metabolism and signal transduction cells that produce small amounts of ROS [94]. However, under inflammatory conditions, the production of ROS, as a mediator of OA disease progression, is significantly increased. In this regard, Lei et al. constructed a bi-dynamic covalently cross-linked hydrogel by cross-linking 3-aminophenylboronic acid modified hyaluronic acid (OHA-PBA) with hydroxyl-containing polyvinyl alcohol (PVA) [54]. Since the phenylboronic acid of OHA-PBA and the hydroxyl group from PVA were able to form a dynamic acylhydrazone bond, it was ROS-responsive and had excellent ROS-scavenging ability, which contributed to the reduction in inflammation and the relief of joint pain and swelling (Figure 8B). Moreover, with ROS scavenging, the borate bond will break, and the hydrogel will gradually transition from the crosslinked state to the free state, from which a sustained release of sodium hyaluronate can be obtained. This controlled release mechanism helps to overcome the problem of rapid metabolism of free sodium hyaluronate, permitting fewer injections and prolonging the therapeutic effect.

In short, the use of hydrogel alone for the treatment of OA is mainly through the properties of the materials used to synthesize the hydrogel. The use of some biomolecules to synthesize hydrogels can both meet their biocompatibility and simulate the living environment of chondrocytes [95], while at the same time making use of the antimicrobial or lubricating properties of these materials, then the repair and regeneration of cartilage is naturally solved. However, the mechanical properties of the regenerated cartilage need to be tested to see if it can support everyday needs when the treatment is applied in the clinic.

### 3.2. HA Hydrogels Loaded with Drugs or Cells

#### 3.2.1. Cell-Loaded HA Hydrogels

Loading cells in hydrogels has been a treatment for OA in recent years because cartilage itself lacks blood vessels and nerves to the extent that it is unable to proliferate cells on its own, and hydrogels can provide a microenvironment suitable for cell survival. Therefore, artificially providing cells and a suitable environment to the defective area can help cartilage repair and regeneration.

**Figure 9:**
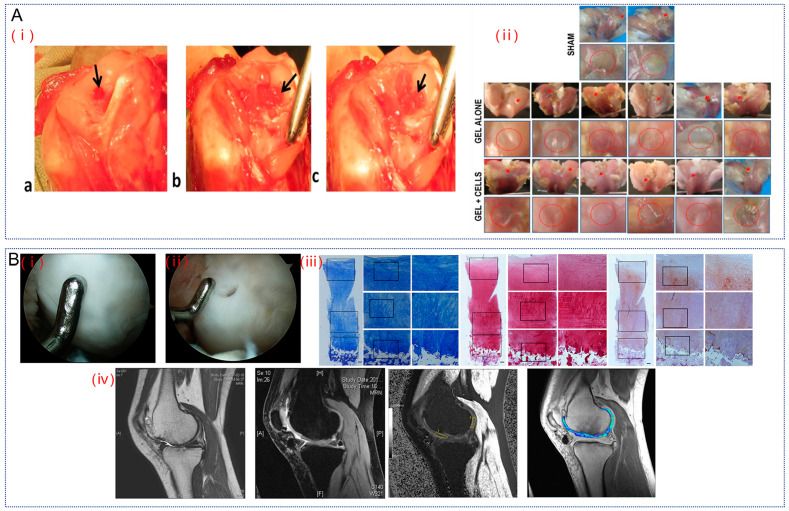
Cell-loaded HA hydrogel for the treatment of OA. (**A**). (**i**) (**a**) Osteochondral defect of rabbit knee joint, untreated (sham operation). Arrow points to the defect site. (**b**) Implant a separate gel at the defect site. (**c**) Allogeneic chondrocyte coated gel was implanted at the defect site. Arrows point to gel/gel + cells at the defect site. (**ii**) General appearance of the joint 12 weeks after implantation. Arrows point to regenerated tissue and circles indicate enlarged areas of regenerated cartilage. Reproduced with permission from ref. [97], copyright, Elsevier. (**B**). (**i**) Arthroscopic examination 1 year after human umbilical cord blood-derived MSC-HA hydrogel composite transplantation reveals good surface reconstruction at the defect site, with smooth surface of the regenerated cartilage and good fusion with the surrounding cartilage. (**ii**) Biopsy samples were taken from the regenerated cartilage. (**iii**) Histological presentation of the regenerated cartilage. Histological analysis of the biopsy samples showed that the staining pattern of the regenerated cartilage was similar to that of normal articular hyaline cartilage. (**iv**) MRI of cartilage regeneration after transplantation of hUCB-MSCs-HA hydrogels at 3 years. Reproduced with permission from ref. [101], copyright, Oxford Academic.

Chondrocyte implantation is the traditional technique for repairing small cartilage defects in young patients [96]. Therefore, Mohan et al. modified HA to HA dialdehyde, whose aldehyde group reacts with the amino group of chitosan via a Schiff base bond to produce a stable gel and encapsulate chondrocytes in the gel [97]. This experiment was performed on rabbit knees with artificial defects, which were divided into a pure gel group and a gel-encapsulated cell group, and after 12 weeks of implanting different gels into the defect site it could be seen that the repaired tissues formed in the sham operation were fibrous and opaque, whereas the tissues treated with the gels had a texture similar to that of the surrounding natural cartilage (Figure 9A). Theoretically, chondrocytes cultured on these gels retained their spherical phenotype and cartilage-specific extracellular matrix. However, although the gels encapsulating the cells initiated a repair process that resulted in the formation of an extracellular matrix that bound to neighboring tissues, the cells encapsulated in the gels did not retain their phenotype for 12 weeks, which resulted in the formation of cartilage that was fibrocartilaginous in nature. Overall, the hydrogel did provide a suitable microenvironment for regenerating hyaline cartilage at the defect site, but the quality of the regenerated cartilage was not significantly improved in the presence of encapsulated chondrocytes [97]. This may be due to the lack of continuous nutrient supply to the defect site leading to cell de-differentiation.

Chondrocytes easily lose their morphology, phenotype and function during in vitro expansion due to their low proliferative capacity, and older chondrocytes exhibit lower biosynthetic and mitogenic activity and weaker response to growth factors as patients age. At present, mesenchymal stem cells (MSCs) are the ideal choice to replace chondrocytes in cartilage tissue engineering due to their strong chondrogenic ability, abundant cell source, low immunogenicity and few ethical problems [98].

It is well known that human mesenchymal stem cells (hMSCs) can be derived from many human tissues, such as bone marrow, cord blood, and adipose tissue [99]. However, the number of MSCs obtained from bone marrow is very limited, and human umbilical cord blood-derived MSCs (hUCB-MSCs) have a higher rate of reproduction, karyotypic stability and greater chondrogenic potential after prolonged amplification compared to BM-MSCs [100]. Therefore, Park et al. performed composite transplantation of hUCB-MSCs and HA hydrogel in seven patients with OA and followed them for up to 7 years to observe their recovery [101]. The drug composed of culture-expanded allogeneic hUCB-MSCs and HA hydrogel for the treatment of cartilage damage in the knee joint is called Cartistem, which has been approved for use in humans since 2012. Mature repaired tissue can be observed at 12 weeks of arthroscopy. Arthroscopic examination after 1 year reveals thick, smooth, white hyaline cartilage at the site of the lesion. Moreover, the regenerated cartilage had a smooth surface and fused well with the surrounding natural cartilage, and no bone formation or overgrowth was observed. Cartilage imaging performed at year 3 showed consistently regenerated cartilage with high GAG content (Figure 9B). Also, during this 7-year follow-up, the participants showed no significant deterioration in pain or function. This demonstrates that the application of a novel drug based on allogeneic hUCB-MSCs is safe and effective for continuous hyaline cartilage regeneration of knee joint with OA.

Recently, the use of 3D-printed dynamic hydrogels in tissue engineering has also emerged. It can be designed to provide oxygen and nutrients to the encapsulated cells to enhance cell survival and differentiation [102], and it also fulfills the need to generate patient-specific implants using computer-aided design. For example, Shi et al. obtained stable 3D-printed dynamic hydrogels acting on OA through the rapid formation of hydrogels by dynamic borate bonding between HA-PBA and PVA, and secondary cross-linking between the acrylate moiety on HA-PBA and the free thiol group from thiolated gelatin [71]. The dynamic properties based on borate bonding allow these hydrogels to have good shear-thinning and self-healing properties, making the hydrogels well-printable, which enables the fabrication of porous lattice structures and meniscus-like scaffolds with high structural fidelity. Such hydrogel scaffolds protect cells from ROS damage and enable stable deposition of extracellular matrix components, including glycosaminoglycans and type II collagen. Shokri et al. used a mixture of HA, gelatin, and elastin to make printable EGH inks for nasal septal cartilage regeneration. The experimental results demonstrated that the EGH scaffolds were able to fuse with the surrounding cartilage, demonstrating the ability to regenerate cartilage defects, and that hydrogel scaffolds loaded with autologous chondrocytes presented better results [103]. Bio-3D printing enables different distribution and arrangement of cells in different layers of the scaffold, which can mimic the anisotropic regional characteristics of natural cartilage [104]. Moreover, bioactive molecules can be added to the biologic links to direct cell differentiation and achieve specific regions of cell morphology and ECM formation [105].

In summary, chondrocytes, although they fit well into the cellular tissue of the damaged area, are already fully developed and can easily de-differentiate without a constant supply of nutrients over a long period of time. In contrast, MSCs have more time to develop and are more compatible with the body’s natural growth. Therefore, HA hydrogels encapsulating MSCs are more suitable for clinical application than simple HA hydrogels.

#### 3.2.2. HA Hydrogel for Encapsulating Drugs

As OA progresses, the ECM component of cartilage will gradually degrade. Therefore, the primary goal of cartilage regeneration is to restore the function of the ECM and to maintain and enhance the cartilage environment to further promote chondrocyte migration and adhesion to establish and maintain cartilage function. To restore the ECM the inflammation first needs to be resolved, and this is achieved by administering medication to the affected area. Current treatment is limited to relieving OA symptoms by taking some analgesics and NSAIDs or intra-articular steroid injections [106]. However, these drugs degrade quickly in the body and need to be administered multiple times, so administering them through a controlled release system is a new breakthrough.

**Figure 10:**
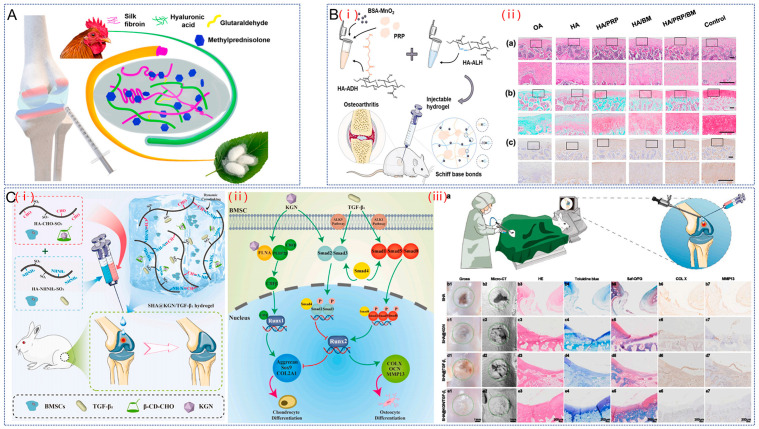
Drug-encapsulated HA hydrogel for the treatment of OA. (**A**). Schematic diagram of the surgical procedure for the formation of an injectable SF/HA hydrogel scaffold and regeneration of cartilage tissue. Reproduced with permission from ref. [55], copyright, Elsevier. (**B**). (**i**) Schematic diagram of the synthesis of HA/PRP/BM injectable hydrogels. (**ii**) Therapeutic effect of each hydrogel treatment group on OA model. (a) HE staining, (b) SO/FG staining and (c) collagen II protein immunohistochemical staining. Reproduced with permission from ref. [56], copyright, Elsevier. (**C**). (**i**) Development and application of SHA@KGN/TGF-β_1_ multifunctional hydrogel. (**ii**) Schematic diagram of the synergistic interaction between KGN and TGF-β_1_. (**iii**) Arthroscopic in situ implantation of various hydrogels for repairing articular cartilage for 8 weeks. Figure (**a**) shows a schematic diagram of arthroscopic ACD repair. Figures (**b1**,**b2**), (**c1**,**c2**), (**d1**,**d2**) and (**e1**,**e2**) are gross and microscopic images after repair. Figures (**b3**–**b7**), (**c3**–**c7**), (**d3**–**d7**) and (**e3**–**e7**) show the staining of each group. Reproduced with permission from ref. [57], copyright, Elsevier.

For drug delivery systems, HA-based hydrogels have been identified as a useful vehicle for drug delivery [107]. Therefore, Phan et al. prepared SF/HA hydrogels by chemically cross-linking HA and lysine residues of filipin protein (SF) through Schiff base formation, and encapsulated methylprednisolone (MP) in the hydrogels to reduce inflammation (Figure 10A) [55]. MP reduces the inflammation of the affected area by inhibiting the accumulation of pro-inflammatory proteins and inflammatory cytokines, while it can promote the release of cytokines from immune cells to enhance the expression of anti-inflammatory proteins, thus providing anti-inflammatory treatment to the affected area. In the SF/HA hydrogel system, the release of MP was prolonged up to 10 days, and the hydrogel had injectable properties to reduce trauma. This experiment demonstrated that HA-based drug-carrying hydrogels can be used as potential biomaterials for cartilage regeneration.

However, because the pathogenesis of OA is more complex, it cannot be treated exclusively through the use of a single functional component. And because of the limitations of OA, intra-articular (IA) injection therapy is gradually becoming a more common treatment option [108,109]. Zhou et al. prepared a HA hydrogel by combining the aldehyde group on OHA with the amino group on HA-ADH via the Schiff base reaction, while bovine serum albumin (BSA)-manganese dioxide MnO_2_ nanoparticles (NPs) dispersed into a HA/platelet-rich plasma (PRP) gel through a Schiff base reaction network, a manganese dioxide nano-enzyme encapsulated hydrogel was prepared [56]. In this hydrogel, MnO_2_NPs can effectively remove reactive oxygen species (ROS); PRP can achieve controlled release of growth factors (GFs), thereby promoting chondrocyte proliferation; serum albumin, as the most abundant protein in blood, can provide nutrients for cartilage repair and regeneration. Moreover, the Schiff base reaction can be generated between the aldehyde group on HA-ALH and the amino group on HA-ADH, BSA, and PRP, which makes the hydrogel self-healing. Due to the pH responsiveness of the Schiff base bond, the encapsulated GFs and BM NPs could be responsively released from the hydrogel network on demand when the hydrogel was exposed to a weakly acidic inflammatory microenvironment. Meanwhile, in vitro experiments confirmed that this integrated hydrogel was more effective in promoting chondrocyte proliferation and protecting chondrocytes from oxidative stress compared with other single-component hydrogels (Figure 10B). Therefore, this multifunctional platform has good potential for application in OA therapy.

#### 3.2.3. HA-Based Hydrogel for Simultaneous Loading of Drugs and Cells

Hydrogels have a therapeutic effect on OA whether they are drug- or cell-loaded. So, would the therapeutic effect of a hydrogel loaded with both drugs and cells be better? With this idea in mind, Gao et al. conducted experiments in which they encapsulated both cells and loaded two drugs in a hydrogel for arthroscopic articular cartilage defect (ACD) repair [57]. They composed solution A of sulfated hyaluronic acid (SHA) modified with aldehyde (HA-CHO-SO_3_), aldehyde-modified β-cyclodextrin (β-CD-CHO), and kartogenin (KGN), and solution B of hydrazine-modified sulfated hyaluronic acid (HA-NHNH_2_-SO_3_) loaded with transforming growth factor β_1_ (TGF-β_1_) and BMSCs, and then directly mixed solutions A and B. A and B solutions were directly mixed to prepare dual-loaded (SHA@KGN/TGF-β_1_) hydrogels. In solution A, KGN pre-complexes with the hydrophobic cavity of β-CD-CHO. After mixing, HA-CHO-SO_3_ in solution A can adsorb TGF-β_1_ in solution B via the sulfonic acid group, while the aldehyde group on β-CD-CHO can directly react with acylhydrazone to load KGN onto the HA-NHNH_2_- SO_3_ network, so that based on the Schiff base reaction between β-CD-CHO and HA-NHNH_2_-SO_3_, KGN was successfully and TGF-β_1_ encapsulated in the hydrogel network (Figure 10C).

Numerous studies have demonstrated that bio inducible factors are critical for chondrogenic differentiation and maintenance of bone marrow mesenchymal stem cells [110]. And TGF-β_1_ is a typical bio inducible factor that is widely used to drive chondrogenic differentiation of bone marrow mesenchymal stem cells [111]. However, TGF-β_1_-induced regenerated cartilage from BMSCs is prone to chondrogenic hypertrophy and even endochondral osteogenesis [112,113]. It has been shown that KGN has demonstrated the potential to promote chondrocyte differentiation and inhibit bone marrow MSC hypertrophy [114]. Therefore, co-delivery of TGF-β_1_ and KGN in an injectable hydrogel may have complementary effects on the enhancement of chondrogenesis and the inhibition of hypertrophy of bone marrow MSCs, thus improving the efficiency of chondrogenesis and counteracting the proliferative tendency induced by TGF-β_1_ [57]. To this end, Gao et al. used a minimally invasive method to create a 4 mm-diameter full-layer articular cartilage defect in the synovial groove of the knee joint and injected different groups of hydrogels into the defective area of the articular cartilage with the assistance of arthroscopy. At 8 weeks postoperatively, the SHA@KGN/TGF-β_1_ hydrogel group showed the best repair results and complete healing of the defect, as shown by the naked eye and micro-CT images [57]. This shows that the synergistic effect of KGN and TGF-β_1_ through sustained release can further support cartilage formation and inhibit the hypertrophy of loaded bone marrow mesenchymal stem cells. The prepared injectable SHA@KGN/TGF-β_1_ hydrogel may be a promising vehicle for stem cell loading and arthroscopic ACD repair.

### 3.3. HA-Based Hydrogels Loaded with Metal Ions

**Figure 11:**
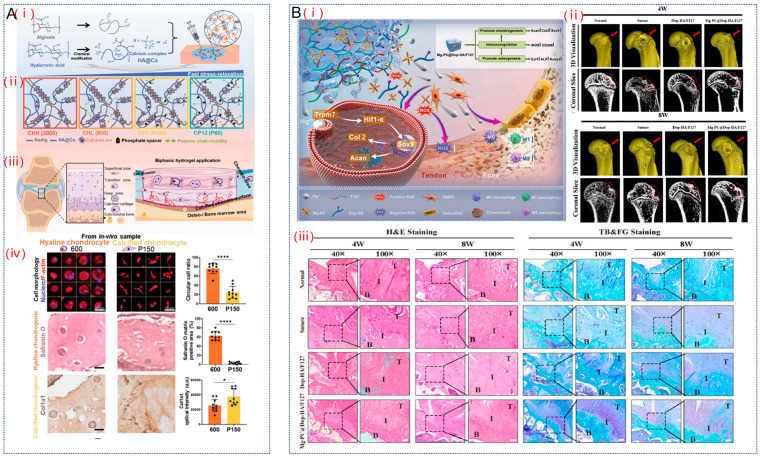
HA hydrogel loaded with metal ions for the treatment of OA. (**A**). (**i**) Chemical structure of prepared calcium hyaluronate complex (HA@Ca) and its injectable hydrogel. (**ii**) Schematic representation of the enhanced remodeling hydrogel mechanism. (**iii**) Schematic and application of HA based biphasic hydrogels for selective hyaline and calcified cartilage regeneration. (**iv**) Nucleic acid and f-actin staining of samples taken from in vivo experiments in a simulated in vivo model of cartilage regeneration to confirm whether phosphorylated HA affects chondrocyte morphology. * *p* < 0.05 and **** *p* < 0.0001 Reproduced with permission from ref. [115], copyright, Wiley. (**B**). (**i**) Mg-PC@Dop-HA/F127 hydrogel repair cartilage by regulating the inflammatory environment and controlling the release of Mg-PC. (**ii**) Reconstruction of the proximal humerus in rats and acquisition of coronal images at 4 and 8 weeks post-surgery using CT technology. (**iii**) Morphological analysis of the newly formed tendon-bone interface tissue after treatment. Reproduced with permission from ref. [58], copyright, KeAi.

In recent years, it has been found that certain metal ions can provide a certain effect on tissue repair and regeneration, so encapsulating metal ions into hydrogels has become a novel way to treat OA. For example, Kim et al. developed an injectable remodeling hydrogel based on HA-calcium composite (HA@Ca) by incorporating calcium or phosphate components into the HA skeleton, which provided stability and mechanical tunability to simulate cartilage (Figure 11A) [115]. This hydrogel was prepared by the technique of phosphorylated HA molecules bound to calcium ions by acid-base reaction first, followed by mixing sodium alginate (NaAlg) with HA@Ca solution. Since calcium ions can move freely within the hydrogel, the two pieces of hydrogel can be integrated within 1–2 min, which is faster for self-healing compared to hydrogel without Ca^2+^.

A calcified cartilage layer exists at the osteochondral interface. This calcified cartilage layer acts as a physical barrier to unwanted vascular invasion and bone growth, which is essential for cartilage defect repair [116]. Thus, the addition of phosphate has a dual benefit: accelerating stress relaxation and fostering a specific environment for calcified cartilage [115]. In contrast, the introduction of HA@Ca regulates the molecular weight and degree of phosphorylation of HA, thereby controlling the remodeling properties of the hydrogel. The stiffness and stress relaxation properties of the polymers were gradually modulated by changing the mobility of the polymer chains through the selection of the type and ratio of alginate and HA@Ca. It was observed that faster relaxation contributed to increased chondrocyte matrix production [115]. Thus, by adjusting the ratio, two hydrogels with different roles can be obtained, both of which can be combined to maintain a strong barrier between cartilage and bone during the healing process, ensuring long-term cartilage integrity and homeostasis in vivo and facilitating a seamless transition from cartilage to bone. The ability of hydrogels to precisely regulate their mechanical and stress-release properties opens up new possibilities for stem cell therapy and tissue regeneration.

Recently, the bioactivity of magnesium ion (Mg^2+^) has garnered significant attention in the field of orthopedic biomaterial research and development due to its potential for promoting bone, cartilage, and periosteum repair [117,118]. Li et al. synthesized magnesium-proanthocyanidin coordinated metal polyphenol nanoparticles (Mg-PC) by a self-assembly process and integrated them into a hydrogel consisting of Dop-HA and F127 [58]. Metal-phenol complexes, formed by the interaction of various metal ions with phenolic molecules, have become a strategy for building drug delivery platforms [119]. Among them, magnesium ions are the metal ligands of proanthocyanidins, and the combination can produce flower-like nanoparticles with a high specific surface area. Procyanidins (PCs) are potent free radical scavengers enriched in grape skins, with anti-inflammatory, antioxidant, and antibacterial effects; M2-type macrophages play a role in pathogen clearance, anti-inflammatory response and metabolism as well as tissue remodeling, whereas the presence of Mg^2+^ induces the differentiation of macrophages to the M2-type, and at the same time, Mg^2+^ can also promote the migration, adherence, proliferation, and chondrogenic differentiation of stem cells in the injury site, and thus promote cartilage differentiation, which in turn promotes the generation of cartilage matrix. Therefore, Mg-PC nanoparticles were covalently crosslinked with the catechol fraction of the hydrogel through ligand bonds, which allowed the hydrogel to continuously and controllably release Mg^2+^ and proanthocyanidins to attract MSCs and progenitor cells, reduce inflammation, and promote macrophage polarization towards the M2 phenotype (Figure 11B).

Overall, this innovative composite hydrogel introduces a new approach to accelerate tissue repair and regeneration while also reducing local inflammatory responses, demonstrating great potential in the field of cartilage regeneration.

## 4. Conclusions and Future Prospects

HA is widely used in cartilage tissue engineering because it has the advantages of good biocompatibility and degradability, and it can promote cell adhesion and proliferation, modulate inflammation, and promote cartilage regeneration [120]. And hydrogel, as a substance with three-dimensional bionic structure, has a wide range of applications in cartilage tissue engineering. Therefore, through the modification of functional groups on the surface of HA, the cross-linking and reaction between HA and other materials can be realized to enhance its biological activity [59,121]. In recent years, with the progress of science and technology, a variety of bioactive materials represented by HA-based hydrogels are gradually being recognized and show a broad application prospect.

In this paper, we focus on the use of dynamic HA-based hydrogels in cartilage tissue engineering. It can be found that different cross-linking methods and different materials can exhibit different functions, so the selection of a suitable hydrogel has a large impact on cartilage repair and regeneration. In addition, since the hydrogel contains more voids, it can be a suitable place for cell proliferation as well as loading drugs into the hydrogel, which makes the hydrogel acting on cartilage injuries have more therapeutic modalities.

However, challenges remain in further translating basic research into broader clinical applications. Firstly, despite the success of chemically or physically cross-linking HA chains to form hydrogels for regenerative medicine, it is currently difficult to be approved for clinical use given the biocompatibility and biosafety [122]. Secondly, although HA hydrogels can be used as functionalized delivery vehicles for bioactive macromolecules or living cells, the cross-linking of the hydrogels, their degradability, their manufacturing dimensions, and the removal of chemical residues during the manufacturing process need to be tightly controlled for clinical applications. Finally, it is also necessary to consider the mechanical properties of HA-based hydrogels, considering the difference between rats or rabbits (the animals used in most of current in vivo studies), as we know, human articular cartilage is subjected to greater pressure than animal cartilage, and whether HA-based hydrogels can withstand this pressure is still open to question. It may be necessary to explore other materials and combine them with HA to improve the mechanical properties of hydrogels [123]. Therefore, minimizing the potential toxicity and improving the biocompatibility, achieving or maintain the long-term mechanical stability should particularly noticed in future via developing novel crosslinking strategies and integrating bioactive cues or agents.

Overall, HA-based dynamic hydrogel systems have yielded results in the application of cartilage repair and regeneration. At the same time, the gradual deepening of the understanding of the relationship between the structural properties of hydrogels, as well as the achievement of novel fabrication techniques, will continue to drive the progress of HA-based hydrogels in biomedical applications. In terms of clinical applications, however, HA-based therapeutics still present a variety of challenges. However, with the urgent need for tissue regeneration therapies and the development of precision medicine, HA hydrogels will have broader clinical applications.

## Figures and Tables

**Table 1:** Materials, crosslinking modes, and characteristics of HA dynamic hydrogels containing different loadings for cartilage repair applications.

Stowage	Materials	Crosslinking Mode	Characteristic	Ref.
not present	oxidized hyaluronic acid (OHA) and N-(2-hydroxypropyl)-3-trimethylammonium chitosan chloride methacrylate (HTCCMA)	Schiff base reaction	Lubrication; Antibacterial; Adhesion	[53]
not present	3-aminophenylboronic acid modified HA and hydroxyl-containing polyvinyl alcohol (PVA)	Phenyl borate bond	Lubrication; Scavenging active oxygen species	[54]
methylprednisolone (MP)	silk fibroin (SF) and HA	Schiff base reaction	Anti-inflammatory; Injectable	[55]
bovine serum albumin (BSA)-MnO_2_ (BM) nanoparticles (NPs)	HA and platelet-rich plasma (PRP)	Schiff base reaction	pH-responsive release; Attenuate the oxidative stress; Promote chondrocyte proliferation	[56]
bone marrow stem cells (BMSCs) and kartogenin (KGN) and transforming growth factor β_1_ (TGF-β_1_)	aldehyde modified SHA (HA-CHO-SO_3_), hydrazide modified SHA (HA-NHNH_2_-SO_3_), and aldehyde modified β-cyclodextrin (β-CD-CHO)	Schiff base reaction	Injectable; Adhesion; Slow-release drug; Promote cartilage differentiation	[57]
magnesium-procyanidin coordinated metal polyphenol nanoparticles (Mg-PC)	dopamine-modified hyaluronic acid (Dop-HA) and F127	hydrogen bond	Injectable; Shape adaptability; Sustainable release; Attracted mesenchymal stem and progenitor cells; Alleviated inflammation; Promoted macrophage polarization towards the M2 phenotype	[58]

## Data Availability

Not applicable.
